# Dietary Fats and Chronic Noncommunicable Diseases

**DOI:** 10.3390/nu10101385

**Published:** 2018-09-30

**Authors:** Hayley E. Billingsley, Salvatore Carbone, Carl J. Lavie

**Affiliations:** 1 Pauley Heart Center, Virginia Commonwealth University, Richmond, VA, 23298, USA; hayley.billingsley@vcuhealth.org; 2 John Ochsner Heart and Vascular Institute, Ochsner Clinical School, University of Queensland School of Medicine, New Orleans, LA 70121, USA; clavie@ochsner.org

**Keywords:** cardiometabolic disease, unsaturated fat, Mediterranean diet, low-fat diet

## Abstract

The role of dietary fat has been long studied as a modifiable variable in the prevention and treatment of noncommunicable cardiometabolic disease. Once heavily promoted to the public, the low-fat diet has been demonstrated to be non-effective in preventing cardiometabolic disease, and an increasing body of literature has focused on the effects of a relatively higher-fat diet. More recent evidence suggests that a diet high in healthy fat, rich in unsaturated fatty acids, such as the Mediterranean dietary pattern, may, in fact, prevent the development of metabolic diseases such as type 2 diabetes mellitus, but also reduce cardiovascular events. This review will specifically focus on clinical trials which collected data on dietary fatty acid intake, and the association of these fatty acids over time with measured cardiometabolic health outcomes, specifically focusing on morbidity and mortality outcomes. We will also describe mechanistic studies investigating the role of dietary fatty acids on cardiovascular risk factors to describe the potential mechanisms of action through which unsaturated fatty acids may exert their beneficial effects. The state of current knowledge on the associations between dietary fatty acids and cardiometabolic morbidity and mortality outcomes will be summarized and directions for future work will be discussed.

## 1. Introduction

Cardiometabolic diseases are estimated to cause over 700,000 deaths per year in the United States (US) and nearly 50% of these deaths are directly related to diet [1,2], although this could be questioned due to so much data being based on potentially flawed food frequency questionnaires (FFQs) [3,4]. Numerous foods, dietary patterns, and individual nutrient consumption have been studied for associations with cardiometabolic disease and its risk factors, but few have received scrutiny as intense and lengthy as dietary fat. As early as 1953, Ancel Keys published a proposed link between dietary fat and cardiovascular diseases (CVD), and five years later, his Seven Countries study began collecting data in an effort to establish a relationship between diet and CVD [5,6]. During the same period, controlled-feeding studies demonstrated a link between increasing saturated fatty acids (SFA) in the diet and increased levels of total cholesterol and, more importantly, of low-density lipoprotein cholesterol (LDL-C), which were known to be associated with incidence of coronary heart disease (CHD) [7]. These controlled-feeding studies, in addition to observational evidence, were relied on as sufficient evidence that a higher total fat and SFA intake leads to an increased incidence of CHD by increasing plasma total cholesterol and LDL-C—the “diet-heart hypothesis” [7,8,9]. In response to this accruing evidence, the very first Dietary Guidelines for Americans in 1980 recommended lowering total fat and SFA [10], which was updated in 1990 to recommend Americans select a low-fat diet, specifically one that consisted of ≤30% total fat and ≤10% SFA of total daily energy [11].

In the decades following these recommendations, total dietary fat decreased in the American diet [12], refined grain intake increased [13], prevalence of type 2 diabetes mellitus (T2DM) continued to rise, and CVD remained the most common cause of death in US adults [14,15]. It is evident that perhaps the most important finding from the Seven Countries Study, that unsaturated fatty acids (UFA) were correlated with a lower risk of CVD in the context of a Mediterranean-style dietary pattern, was not properly communicated and implemented [16]. Evidence in favor of higher UFA consumption within a healthy dietary pattern has continued to accumulate and in 2015, the Dietary Guidelines for Americans was published for the first time in 35 years without a recommended limit on total dietary fat consumption while continuing to promote UFA consumption [17].

This review will discuss the evidence behind the recommendation of the low-fat diet for the past few decades as well as the evidence of UFA intake for cardiometabolic health by first reviewing morbidity and mortality outcomes. Finally, we will discuss the potential underlying mechanisms through which fatty acids may affect overall cardiometabolic health.

## 2. Methodology

To identify the works used in this critical review, the comprehensive electronic literature search using PubMed and Google Scholar included the use of the following key words and their combination: “Low-fat diet”, “high-fat diet”, “Mediterranean diet”, “fatty acids”, “mortality”, “cardiovascular disease”, “weight gain”, “weight loss”, “type II diabetes”, “insulin resistance”, “blood pressure”, “heart failure”, “dyslipidemia”, and “cancer”. All works meeting the subject matter were considered including observational cohort studies, randomized controlled trials, reviews, meta-analyses, and editorials, in animals and adult humans. Preference was placed on the most recent papers with the exception of those providing historical context to subject matter.

## 3. Introduction to Dietary Fat Nomenclature

Dietary fat can be broadly broken down into 3 broad subtypes—UFA, SFA and trans fat. UFA can be further divided into monounsaturated fat (MUFA) and polyunsaturated fats (PUFA). Numerous unique fatty acid molecules belong to each subtype of fat, sometimes with divergent effects due to their unique properties as well as the foods they exist within. Additionally, complex mechanisms exist within the body to process consumed nutrients and knowledge continues to accrue regarding the metabolism of dietary fat. A recent example is the recognition of the skeleton and particularly bone marrow as major contributors to fatty acid metabolism [18]. SFA exist in small amounts in many diverse sources of dietary fat, but animal products such as dairy (milk, butter, cheese) and meat are higher in SFA than most plant oils. Notable exceptions are tropical plant oils such as palm and coconut oil, which are also rich sources of SFA. Major sources of SFA include lauric acid (C12:0), myristic acid (C14:0), and palmitic acid (PA) (C16:0) which all increase LDL-C, and stearic acid (C18:0), which does not [19]. There has been some suggestion that PA (C16:0) found in both animal products and palm oil has particularly deleterious effects on cardiometabolic health, such as increased inflammation, oxidative stress, and impaired nitric oxide and insulin signaling, but due to food sources being high in multiple SFAs and endogenous production of PA by the body it is somewhat difficult to distinguish divergent effects [20,21]. Different sources of SFA may have slightly different risk profiles—recent data has suggested that dairy fat in particular may be neutral or even beneficial on cardiovascular (CV) outcomes [22,23]. This, however, seems to be related to the relative risk compared to refined carbohydrate: animal meat sources of SFA and dairy fat still increases atherosclerotic lipoproteins and replacement with UFA results in a decreased risk of CHD [24]. MUFA are also found in meat and dairy, but rich plant sources of MUFA include extra-virgin olive oil, canola oil, and some other plant oils such as rapeseed and high-oleic sunflower and safflower. The major MUFA present in Western diets and referenced in this review is oleic acid (C18:1*n*-9), however, some populations have a high intake of rapeseed oil, and therefore a high intake of erucic acid (c22:1*n*-9) [19]. PUFA are found in nuts and seeds, cold-water fish, and plant oils such as soybean and flaxseed. PUFA are divided in two categories, *n*-6 and *n*-3, based on the position of the first double bond from the omega end of the fatty acid. The primary source of PUFA in the diet is linoleic acid (LA) (C18:2*n*-6), an *n*-6 fatty acid [19]. Plant sourced *n*-3 alpha linolenic acid (ALA) (C18:3*n*-3) is present to a lesser degree, and even less prevalent are the marine *n*-3 fatty acids eicosapentaenoic acid (EPA) (C22:5*n*-3) and docosahexaenoic acid (DHA) (C22:6*n*-3) [19]. Though *n*-3 and *n*-6 PUFAs will be discussed separately in this review, both have been shown to be beneficial to human health when consumed in food sources and *n*-6 PUFAs are no longer considered to be inflammatory [25]. Trans fats occur to a very small degree naturally but mostly exist in the form of engineered fats in highly-processed convenience foods or fast food. Engineered trans fats have been established as harmful to human health and widespread efforts to remove sources of trans fat from the global food supply are ongoing [19,26].

### 3.1. Low-Fat Diet and CVD

In spite of the decades of guidelines recommending a low-fat diet, few trials have explored the long-term implementation of a low-fat diet and the resulting morbidity and mortality outcomes. The largest trial examining the cardiometabolic effects of the low-fat diet was the Women’s Health Initiative Dietary Modification Trial (WHI) which tested the effects of a low-fat diet targeted at reducing the percentage of daily calories from dietary fat to 20%, while increasing servings of grains, fruits, and vegetables [27]. Over 48,000 postmenopausal women were randomized to either a low-fat intervention (40% of participants) or a control arm with minimal dietary interference (60% of participants) [28]. The women in the intervention arm received 18 group counseling sessions focused on dietary modification in the first year and then quarterly for the reminder of the trial [28]. A prescription for grams of daily dietary fat was given to each participant but no weight loss or physical activity goal was specified or encouraged [29]. Subjects in the control group received no dietary guidance but regular clinic visits [28]. After an average of 8.1 years follow up, the intervention group successfully decreased dietary fat intake by an absolute 8.2% from a baseline of 32%. No difference in the rate of CHD, stroke, or CVD was found between the intervention group and the control group, as recorded in Table 1. Although the women decreased total fat by 8.2%, SFA intake only decreased by an absolute 2.9% to a mean of 9.5% calories from SFA in the intervention group, which was significantly above the indicated target of 7% defined at the begin of the study. Although this aligns with the World Health Organization and American Dietary Guidelines 2015–2020 recommendations to reduce SFA to below 10% of total calorie intake in the general population, this recommendation did not result in improved clinical outcomes in the WHI study. Of note, the women enrolled in the WHI trial were post-menopausal and overweight with a mean body-mass index (BMI) of 29 kg/m^2^, and were perhaps at a greater CVD risk compared to the general population [17,27,30]. The American Heart Association recommends that individuals with a high LDL-C reduce SFA to 5–6% of daily calories, which may have applied to many of the individuals who developed CVD events during the trial [31]. 

A recent analysis explored deeper into the neutral CVD outcomes of the WHI trial, examining the difference in CVD disease risk modification for those in the low-fat intervention group [32]. This analysis divided the women into three strata: subjects with baseline CVD, normotensive subjects without baseline CVD, and hypertensive subjects without baseline CVD [32]. Interestingly, the results diverged: in subjects with a prior history of CVD randomized to the low-fat intervention, risk was significantly increased for composite CHD as well as all causes of death [32]. In contrast, normotensive women without a history of CVD had a significantly reduced risk of CHD, which was overshadowed by an increase in stroke risk, particularly ischemic stroke risk [32]. No difference in disease risk was seen in hypertensive women without a history of CVD [32]. It is important to note that CHD was a secondary outcome for the WHI trial, while stroke risk was not a designated primary or secondary trial outcome. The authors of the analysis attribute the adverse CHD outcomes in the subjects with prior CVD to alterations in statin use and the increased stroke risk seen in normotensive women without baseline CVD to possible multiple-testing bias [32]. The findings of this analysis are inconclusive but do bring up a disturbing possibility that the low-fat diet is not just neutral on cardiometabolic outcomes, but could potentially be harmful.

### 3.2. Low-Fat Diet and Metabolic Diseases

The WHI investigators also tracked the incidence of T2DM throughout the length of the trial [29]. They hypothesized that a low-fat dietary pattern, even in the absence of weight loss or exercise, would reduce T2DM incidence [29]. Type I diabetes mellitus was an exclusion factor for the trial and subjects with T2DM at baseline were excluded from this analysis [29]. Incidence of T2DM was self-reported and based on medication reconciliation at regular 6-month visits throughout the trial as only around 5% of the over 45,000 women included in this analysis had regular lab work drawn as part of the trial for a confirmative diagnosis [29]. Participants did not differ in risk factors for T2DM at baseline [29]. After 8.1 years, as shown in Table 1, there was no difference in incidence of self-reported T2DM between subjects in the low-fat intervention and control groups [29,33]. Greater reduction of dietary fat from baseline was initially associated with reduced T2DM but this was no longer significant after controlling for weight loss [29].

Low-fat diets were once considered to be superior weight loss tools as fat, at 9 calories per gram, is more energy-dense than carbohydrate and protein, so reducing fat intake appeared to be an effective way to cut calories from a person’ diet [34]. In contrast, evidence suggests that low-fat diets are equivalent, at best, to other diets for weight loss, not superior [35,36,37,38,39]. A recent meta-analysis that included randomized controlled trials (RCTs) with over one year of follow up concluded that low-fat dietary trials did not result in greater weight loss when compared to higher fat interventions of similar intensity as measured by the time, individual attention, and program materials given by the investigative team to participants [37]. When weight loss was the goal, higher fat, lower-carbohydrate diets actually resulted in greater weight loss over time [37]. A lack of difference between the two diets in terms of weight loss was recently demonstrated in the high-profile Diet Intervention Examining the Factors Interacting with Treatment Success (DIETFITS) trial, which examined the effects of genotyping and insulin response on weight loss in 609 overweight and obese individuals randomized to a low-fat or low-carbohydrate diet [38]. The DIETFITS intervention employed intensive counseling and education over a 12 months period to both groups, but at the conclusion of the trial, no difference in weight loss was seen between individuals randomized to a low-fat or low-carbohydrate diet, regardless of genotype or insulin-response at baseline [38].

In summary, low-fat dietary patterns do not seem to aid in the prevention of CVD or T2DM, and are not superior to other diets in terms of weight loss. Continued promotion of the low-fat diet is ineffective at preventing cardiometabolic disease at best, and exploratory findings cannot exclude harms. 

### 3.3. High-Fat Diet and CVD

In the last decades, strong evidence has emerged to suggest a diet higher in UFA in the context of a healthy dietary pattern holds strong promise in the prevention and treatment of cardiometabolic disease. This is exemplified in a traditional Mediterranean diet (MedDiet) which is best known as the traditional dietary pattern consumed in Greece, southern Italy and the island of Crete, first brought to the attention of the nutrition scientific community by Ancel Key’s Seven Countries Study [16,40]. Briefly, the MedDiet is a diet rich in fruits, vegetables, whole grains, legumes and UFA, principally from extra-virgin olive oil (EVOO) and nuts. Other features of the MedDiet include the consumption of fish and poultry in moderate amounts, red wine with meals, and low consumption of red meat and refined carbohydrates compared to other Western societies. The Prevención con Dieta Mediterránea (PREDIMED) trial, first published in 2013 [41] and retracted and subsequently republished with corrections in 2018 with similar results [42,43], tested a Mediterranean diet (MedDiet) supplemented with nuts or EVOO against a low-fat control diet in nearly 7500 individuals at high CVD risk to assess prevention on a primary composite endpoint of nonfatal myocardial infarction (MI), stroke, and death from CVD [41,42,43]. This multicenter randomized controlled trial performed in Spain assigned patients 1:1:1 to a MedDiet plus at least 4 tablespoons of EVOO, a MedDiet with 30 g of mixed nuts per day, or a low-fat control diet [42,44]. Notably, no caloric restriction was recommended, and at conclusion of the trial after an average of 4.8 years follow-up the MedDiet groups were consuming 42% total daily energy from fat while individuals in the relatively low-fat control group were consuming around 37% [45]. Patients in the MedDiet groups received group and individual counseling by a dietitian at baseline and then quarterly for the remaining duration of the study [42]. The subjects assigned to the low-fat diet group were initially only given a leaflet with advice on adopting a low-fat diet, but midway through the trial were transitioned to quarterly dietary counseling as well [42].

Adherence to a MedDiet using a 14-point dietary questionnaire was assessed at baseline in all subjects and quarterly thereafter in the MedDiet groups [42]. Control subjects filled out a 9-point dietary questionnaire at dietary counseling sessions assessing their adherence to a low-fat diet [42]. The planned duration of the trial was 6 years but an interim analysis at 4.8 years demonstrated sufficient evidence for benefit to terminate the trial early [46]. In fact, both MedDiet groups demonstrated a 30% relative risk reduction for the primary endpoint compared to their control counterparts [42], as shown in Table 1. This may have been largely driven by a reduction in stroke risk—the combined MedDiet groups demonstrated an impressive 42% relative risk reduction, as seen in Table 1, while death from CVD and nonfatal MI were non-significant independently [42].

The benefit of the relatively high fat MedDiet was not limited to CVD. Although fear of weight gain exists with a higher-fat diet, after 5 years of follow up all three groups showed a weight reduction from baseline and the MedDiet group supplemented with EVOO demonstrated greater weight reduction compared to control participants [47]. Furthermore, both MedDiet groups exhibited a lower increase in waist circumference during the trial than the control group [47,48]. Another analysis of the PREDIMED examined development and reversal of metabolic syndrome (MetS), defined under the integrated International Diabetes Federation/American Heart Association/National Heart, Lung and Blood Institute standards, and found that while there was not a difference between groups in risk of developing MetS, reversal from baseline MetS was more likely in the MedDiet groups compared to control [49]. Additionally, participants in the EVOO group were more likely to have developed a decrease in fasting plasma glucose from baseline [49]. Remarkably, after a median follow-up of 4 years, incidence of new-onset T2DM was reduced by 53% in the two MedDiet groups compared to the control [50,51] (Table 1).

Prevention of heart failure (HF) was also explored in the PREDIMED, but while levels of HF prognostic biomarkers (i.e., NT-proBNP) were reduced in the MedDiet groups in one analysis [54], incidence of new-onset HF was not lower in the MedDiet groups than the control group, perhaps due to a small number of events making the study likely underpowered to investigate the effects on new-incident HF [55]. A recent prospective cohort study, Mediterranean Diet in Acute Heart Failure (MEDIT-AHF), explored secondary prevention potential of the MedDiet for patients who had already experienced acute HF (AHF) [56]. Individuals were enrolled in this study in the emergency department (ED) setting and were administered the 14-point dietary questionnaire used in PREDIMED to assess adherence to the MedDiet [56]. The primary outcome of the MEDIT-AHF trial was all-cause mortality at the end of the trial, secondary outcomes consisted of ED visits for HF, hospitalization due to HF, all-cause mortality, and a composite of the three secondary outcomes if one was present [56]. After an average follow up of 2.1 years, ED visits, all-cause mortality, and the combined variable were not different between adherent and non-adherent individuals, however hospitalization was significantly lower in individuals adhering to the MedDiet [56]. It should be noted that both individuals with HF with a preserved ejection fraction (HFpEF) and HF with a reduced ejection fraction (HFrEF) were enrolled in this study. HFpEF is a condition that currently lacks effective clinical therapies and therefore the reduction in hospitalization seen in this study has important implications for future work [57]. Recently, higher consumption of UFA was correlated with greater cardiorespiratory fitness (CRF) as well as better cardiac diastolic function and body composition in a cohort of obese HFpEF patients [58]. The investigators have initiated a novel pilot study in obese HFpEF patients (NCT03310099) exploring the capabilities of UFA supplementation to improve CRF, metabolic flexibility and glucose tolerance.

### 3.4. High-Fat Diet and Metabolic Diseases

Recently, the results of the Prospective Urban Rural Epidemiological (PURE) study, a prospective cohort of over 135,000 individuals from 18 different countries, called into question whether the type of fatty acid consumed matters in the human diet [59]. Briefly, PURE collected dietary intake through FFQs and followed participants for an average of 7.4 years. Primary outcomes were designated as major CVD events and total mortality. At analysis, high carbohydrate intake was associated with an increased risk of total mortality but was not associated with CVD events or mortality [59]. Total fat and each category of fats (SFA, MUFA and PUFA) were associated with lower risk of total morality but not MI or overall CVD mortality [59]. The investigators stated they were unable to analyze trans fatty acids [59]. SFA alone were associated with lower risk of stroke [59]. The findings of PURE are in direct opposition to current guidelines, which recommend that SFA should be limited in the diet to help prevent CVD [17,60,61]. It is possible in the PURE cohort that FFQs estimated UFA mostly from food sources not deriving from vegetable oils and missed or underestimated vegetable oils [62]. Vegetable oils are major sources of UFA that mostly lack SFA, while animal sources of UFA are also rich sources of SFA [62,63]. It is possible that analyses of the trial, which take different dietary sources into account, could show divergent results between the consumption of different fatty acids.

**Figure 1:**
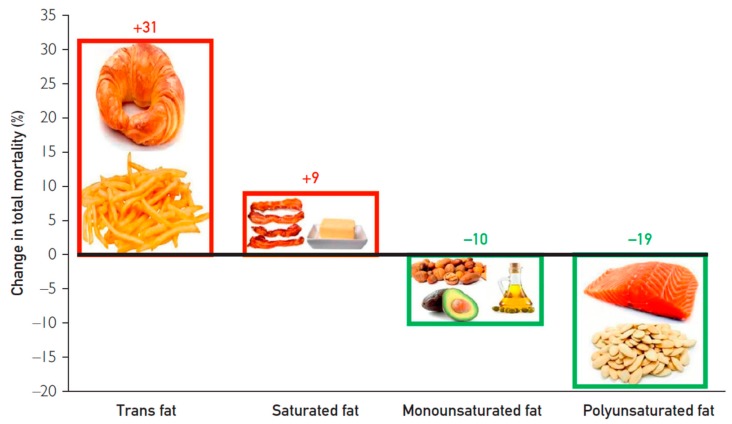
Substitution of different fatty acids for carbohydrate in the Nurse’s Health Study (NHS) and Health Professional’s Follow Up Study (HPFS). Hazard ratios for total mortality by replacing carbohydrates with specific dietary fats. Used with permission from O’Keefe et al. [65].

A recent analysis of the Nurse’s Health Study (NHS) and Health Professional’s Follow Up Study (HPFS) in a cohort of similar size to PURE (>126,000), which also utilized FFQs in a prospective cohort design, illustrated careful comparison of the effects of different fatty acids consumption on clinical outcomes [64]. Similar to what was found in PURE, total dietary fat consumption was inversely associated with total mortality when substituted for total dietary carbohydrates [64]. The authors then performed substitution analysis of different macronutrients [64]. When SFA and trans fatty acids were substituted for total carbohydrate, a higher risk of mortality ensued, but when PUFA and MUFA were substituted for carbohydrate, as seen in Figure 1, a significant reduction in mortality was observed. Likewise, when PUFA and MUFA were substituted for 5% of energy from SFA, a 27% and 13% reduction in mortality was observed [64]. When trans fatty acids were substituted for 2% of energy from SFA, a 16% mortality increase followed [64].

The results of the PURE study also called into question the ideal percentage of calories from carbohydrates vs. protein and fat, as high carbohydrate intake was associated with increased risk of total mortality [59]. The composition of a very low-carbohydrate diet or “ketogenic diet” varies in the literature, making up from <30–130 g of carbohydrate per day [66]. In a true ketogenic state, reserves of glycogen are depleted over the course of a few days on extremely low carbohydrate intake, and the liver begins producing ketone bodies as an alternate fuel source for the central nervous system [66]. In human subjects, positive cardiometabolic effects have been seen, including improved lipid panels and glucose metabolism, but concerns have been raised over the lack of long-term data, high intakes of saturated fat and/or protein, and maintaining adherence to a strict dietary pattern [66]. Additionally, a recent analysis suggested that both low (<40% daily calories from carbohydrate) and high (>70%) carbohydrate diets were associated with a greater risk for all-cause mortality [67]. The lowest mortality occurred with a diet composed of 50–55% of calories from carbohydrate [67]. Importantly, low-carbohydrate diets varied in risk profile—when carbohydrate was replaced by a diet high in plant-based protein and fat, a lower mortality risk was observed, but when calories from carbohydrate were replaced by animal fat and protein, an increased risk of all-cause mortality was observed [67]. Notably, participants in the PREDIMED consumed around 42% of their energy from carbohydrate at baseline, and MedDiet groups slightly decreased intake, while control participants slightly increased. Future work should focus on the effects of a plant-based ketogenic diet (KD) vs. animal-based KD as well as modulating carbohydrates (low vs. moderate) in a high-UFA Mediterranean dietary pattern.

## 4. Potential Mechanisms of Action of Fatty Acids on Cardiometabolic Disease Risk Factors

The cardiometabolic risk reduction observed with a diet higher in UFA is likely multifaceted. Evidence suggests that a diet higher in UFA may affect multiple related risk factors for cardiometabolic disease, including blood pressure (BP), weight maintenance, blood-glucose levels, blood lipids, and inflammation [68]. Though the discussion below focuses on independent mechanisms of improvement for each risk factor, these improvements may also be achieved by the nature of their relation to other risk factors (i.e., weight loss and blood pressure improvement).

Hypertension is responsible for more deaths from CVD in the US than any other modifiable risk factor. Consuming a diet rich in UFA may help lowering BP modestly, by less than 5 mmHg each in systolic and diastolic pressure [69,70], while SFA have an unclear relationship with BP [25]. The mechanism for this reduction is not fully established and may differ between different sources of UFA. Particularly for EVOO, the BP reduction induced may be mediated by OA (C18:1*n*-9) [71], the main fatty acid of EVOO. In pre-clinical studies, administration of EVOO actually altered membrane lipid structure by increasing the amount of OA (C18:1*n*-9) in the cell membrane, which altered G-protein mediated signaling and reduced BP in rats [72].

Nuts may also contribute to the BP-lowering effects of UFA, however, the effects seem to differ, perhaps due to the varying nutrient profile of different nuts. In a meta-analysis including 21 randomized-controlled trials with BP as the primary or secondary outcome, pistachios, but not other nuts, were significantly associated with a significant reduction in systolic BP (SBP) and diastolic BP(DBP) [73]. The authors postulated this could be due to their higher amount of MUFA (particularly OA (C18:1*n*-9)), antioxidants, and the amino acid arginine—a precursor to nitric oxide (NO), a potent vasodilator [74]. A meta-analysis of *n*-3 fatty acids, EPA (C22:5*n*-3) and DHA (C22:6*n*-3) and from food sources and supplementation found that SBP and DBP was lowered in both hypertensive and non-hypertensive subjects [75]. Multiple mechanisms have been suggested for this effect, predominantly relating to endothelial improvements. A recent retrospective analysis of a double-blind, placebo-controlled crossover study supplementing EPA (C22:5*n*-3) and DHA (C22:6*n*-3) in adult subjects attempted to isolate the hypotensive mechanisms of these fatty acids. Though there were no changes in endothelial function or BP in the overall group, SBP was lowered in individuals found to have systolic hypertension [76]. As for the mechanism of action, results demonstrated no effect of adhesion molecule expression in the endothelium or improvement in microvascular function [76]. The investigators also examined the effect of the eNOSrs1799983 gene, associated with lower circulating NO and CVD incidence, and found no treatment x genotype effect leading to speculation that the hypotensive response is independent of the effects of NO [76]. Overall, the effects of different sources of UFA on BP appear to be modest. However, a diet rich in UFA from multiple sources may produce a greater additive effect and subsequent protection against CVD.

Diverging effects on lipid panels are perhaps the one of the earliest and most often cited properties of dietary fats on cardiometabolic health. The National Lipid Association recommends that UFA should be substituted for SFA in lieu of carbohydrates for greater lowering of atherogenic cholesterol levels [77]. The expert consensus was that oils rich in *n*-6 PUFA lower atherogenic cholesterol levels more effectively than MUFA based on controlled feeding trials [77]. However, fatty acids exist within different food sources creating complex effects on lipoproteins. MUFA, especially as part of EVOO, has protective effects against oxidation of LDL-C and high-density lipoprotein cholesterol (HDL-C) as well as promoting an increase in HDL-C [78]. Nuts have varying nutrient profiles and therefore a likely variable effect on lipoproteins [79]. A pooled analysis of 25 intervention trials demonstrated that dietary interventions consisting of nuts alone reduced total cholesterol, LDL-C, and the ratio of LDL-C to HDL-C, but had no effect on HDL-C [80]. The investigators found that this was dose-responsive with higher nut consumption leading to a greater reduction in TC and LDL-C [80]. In addition to UFA, nuts are a source of dietary fiber and plant sterols, which may help reduce cholesterol absorption at the gut level. Lastly, the *n*-3 fatty acids EPA (C22:5*n*-3) and DHA (C22:6*n*-3) have a well-recognized lowering effect on triglycerides (TGs) [81,82]. This reduction in TGs is possibly mediated by multiple mechanisms, including a reduction in circulating fatty acids by increased beta oxidation/decreased lipogenesis, decreased TG synthesizing enzymes, and increased phospholipid synthesis [82]. Again, sources of UFA provide varying effects and mechanisms by which they improve lipid panels and therefore reduce cardiometabolic risk. It is likely the best result is achieved by consuming a diet rich in multiple different sources of UFA.

Weight gain in adulthood is a major risk factor for cardiometabolic disease and some evidence has demonstrated that low-fat diets are not superior to higher-fat diets in terms of weight maintenance or loss [35,37,38,47]. Results from the PREDIMED discussed above suggest that a diet relatively high in UFA may be protective against weight gain [47]. A recent analysis of the European Prospective Investigation into Cancer and Nutrition (EPIC) cohort of over 375,000 European participants corroborated an association between nut consumption and protection against weight gain [83]. The investigators demonstrated that for every 15 g of additional nut consumption per day, participants gained slightly less weight, average of −0.04 kg over 5 years compared to their counterparts, which corresponded to a small but significant 2.5% reduction in body weight increase [83]. Additionally, individuals in the highest quartile of nut consumption who were of a normal weight or overweight at baseline had a 5% lower chance of moving up a body-mass index (BMI) category [83]. Strong evidence is available to indicate that nuts, which contain 50% calories from fat, at the very least do not contribute to increased adiposity [84] and may aid in weight maintenance [47,83]. Nuts likely exert their protective effects against weight gain through several different mechanisms including the effects of the dietary fiber, protein, and fat on satiety, and incomplete absorption of fat at the intestinal level from physical structure [84]. EVOO also demonstrated a protective effect against weight gain and central adiposity during the PREDIMED trial [47]. This was previously demonstrated in the 7368-subject SUN prospective cohort in Spain [85]. In this cohort, participants with the lowest EVOO consumption at baseline who did not increase consumption during the 28.5-month follow-up period gained the most weight [85]. The lowest weight gain was demonstrated in individuals with moderate baseline consumption who increased EVOO during follow up [85]. The mechanisms behind the protective effects of EVOO on weight maintenance are not well established but could be due in part to lowered action of stearoyl-CoA desaturase 1 (SCD1), an obesogenic enzyme that catalyzes the production of MUFA from SFA [86]. Moreover, MUFA can slightly increase resting metabolic rate [87] and physical activity [88], which are, in turn, associated with weight loss in absence of major changes in food intake. Pre-clinical studies also support the beneficial effects on body weight of high-UFA diet, independent of caloric intake [58]. Overall, the evidence shows that nutrient-rich foods high in UFA do not increase the risk of weight gain and may be even protective against increased adiposity. Since 1980, prevalence of T2DM worldwide has doubled in men and increased by 60% in women [89]. Around 8–9% of the world’s adult population now suffers from diabetes, and 8–95% from T2DM, which is heavily associated with lifestyle factors [89]. The scale of this epidemic underlines the importance of the PREDIMED sub-analysis that demonstrated new-onset T2DM was reduced over 50% in the higher-fat MedDiet groups compared to the low-fat control [50,51]. The authors suggested this may be due to the effects of lowered chronic systemic low-grade inflammation—an early sub-analysis showed significantly lowered markers of inflammation including Interleukin-6 (IL-6), soluble intercellular adhesion molecule-1 (ICAM-1), and vascular cell adhesion molecule (VCAM-1) in the MedDiet groups [90]. Additionally, C-Reactive Protein (CRP) was reduced in the EVOO MedDiet group [90]. The anti-inflammatory effects of EVOO and nuts are multi-factorial and may include enhancement of the body’s endogenous antioxidant defenses, protection against weight gain for individuals in the MedDiet groups and the rich source of polyphenols EVOO provides [91]. Additionally, intake of EVOO with a meal has been shown to affect glycemia by improving insulin sensitivity and increasing glucagon like peptide-1 [92].

However, despite the impressive findings of the PREDIMED, evidence associating total dietary fat and subtypes of dietary fat has been inconsistent, which may be due to the differing food sources of fatty acids [93]. For example, *n*-3 FA supplementation is not recommended by the American Diabetes Association due to lack of demonstrated benefit, but the organization continues to recommend the intake of marine fish rich in *n*-3 for more general health benefits [94]. A meta-analysis and systematic review of 102 trials and over 4200 subjects enrolled in controlled feeding trials demonstrated overall beneficial effects of UFA on glucose-insulin homeostasis [95]. In a replacement analysis of 5% of calories, PUFA and MUFA were associated with improved HOMA-IR and HbA1c when replacing carbohydrate or SFA [95]. PUFA improved insulin secretion capacity whether the PUFA replaced carbohydrate or SFA or MUFA [95]. Only the replacement of SFA with PUFA reduced fasting glucose levels [95]. When SFA or PUFA was substituted for carbohydrate, fasting insulin was reduced but substitution of SFA for carbohydrate resulted in a significantly increased c-peptide in several trials [95]. Longer-term randomized-controlled trials supplementing UFA in individuals at risk for T2DM are needed.

## 5. Discussion and Conclusions

Preventing cardiometabolic disease with targeted lifestyle therapy such as evidence-based nutrition interventions must be made a global health priority [96]. There is a lack of evidence demonstrating that a low-fat diet is protective against the development of cardiometabolic disease [27,29,32,33]. Evidence continues to accrue that a diet higher in fat, particularly of UFA, especially in the context of a Mediterranean style dietary pattern may be protective against multiple risk factors for cardiometabolic disease [47,49,90,97]. Most importantly, evidence indicates that improvement in these intermediate markers does translate to prevention of CVD and T2DM in individuals consuming a higher UFA diet [42,50].

It should be noted that while a low-fat diet is not effective in the prevention of cardiometabolic disease, the WHI did demonstrate a reduction in death after breast cancer in the low-fat intervention group versus the control during the trial and throughout the 16.1-year follow up period [52]. No significant reduction in breast cancer incidence was observed [52], as seen in Table 1. Breast cancer incidence was also analyzed in the PREDIMED [53]. During the 4.8 years of follow up, as shown in Table 1, incidence of breast cancer was significantly reduced in both MedDiet groups—the released analysis did not include mortality outcomes [53]. Further research is needed to directly compare a healthy low-fat dietary pattern with a higher-fat MedDiet pattern to determine which pattern is superior in preventing breast cancer as well as mortality during and after treatment.

The PREDIMED was conducted in Spain, a country where a Mediterranean dietary pattern rich in UFA is commonly consumed—EVOO is the main dietary fat source in the Spanish population studied [98]. Interest in replicating the PREDIMED in a non-Mediterranean country is substantial. An expert working group has thoroughly explored the necessary resources, accommodations, and anticipated problems investigators may face in attempting to replicate the PREDIMED in the US [98]. Compared to the PREDIMED participants at baseline, the Mediterranean diet adherence score (MEDAS) of US participants estimated from the National Health and Nutrition Examination Survey is substantially lower [98], as seen in Table 2. Importantly, whether the supplementation of nuts and EVOO works in the context of a non-Mediterranean dietary pattern such as in United States is unknown [99]. The working group suggested that a goal of the trial would be to increase the MEDAS score of the US modestly and not to the level of Spanish participants which they felt would be very difficult to achieve and perhaps maintain [98], as seen in Table 2. 

In summary, a relatively high-UFA diet, especially in the context of the MedDiet, decreases risk factors as well as morbidity and mortality related to CVD. In contrast, there is no evidence that a low-fat diet leads to lower morbidity or mortality related to cardiometabolic disease. Randomized controlled trials are warranted to test the effects of UFA-supplemented MedDiet in diverse global populations on risk factors and outcomes of T2DM and CVD.

## Figures and Tables

**Table 1:** Cardiometabolic outcomes comparison of the Prevención con Dieta Mediterránea (PREDIMED) and Women’s Health Initiative Dietary Modification Trial (WHI) trials.

Outcome of Interest ^1^	WHI (Low-Fat Diet)	PREDIMED (High-Unsaturated Fat Diet)
Composite Cardiovascular Outcome ^2^	0.94 (0.86–1.02)	0.70 (0.55–0.89)
Non-fatal myocardial infarction	0.91 (0.80–1.04)	0.80 (0.53–1.21)
Nonfatal Stroke	1.02 (0.90–1.17)	0.58 (0.42–0.82)
Type 2 Diabetes Mellitus Incidence	0.96 (0.90–1.03)	0.47 (0.26–0.87)
Breast Cancer incidence	0.91 (0.83–1.01)	0.49 (0.25–0.94)

^1^ Data are presented as hazard ratio and 95% confidence interval. ^2^ For WHI: myocardial infarction, coronary heart disease, death or revascularization. For PREDIMED: non-fatal myocardial infarction, cardiovascular death or non-fatal stroke. Hazard ratios and confidence intervals for the WHI versus the PREDIMED on primary prevention of cardiovascular, metabolic and cancer-related endpoints [27,29,42,50,51,52,53].

**Table 2:** Dietary pattern comparison of PREDIMED MedDiet groups and U.S. average intake.

	Mediterranean Diet + Extra-Virgin Olive Oil	Mediterranean Diet + Nuts	NHANES 2011–2012	Change Needed
*Nutrient intake*				
Energy, kcal/day	2172	2229	2141	
Carbohydrate, % Energy	40	40	48	Decrease (type matters)
Protein, % Energy	16	16	16	
Total fat, % Energy	41	42	34	Increase (type matters)
Saturated fat, % Energy	9	9	11	Decrease
Monounsaturated Fatty Acids, % E	22	21	12	Increase
Polyunsaturated Fatty Acids, % Energy	6	8	8	None
α-Linolenic acid, g/day	1.3	1.9	Not Available	Not Available
Marine *n*-3 fatty-acids, g/day	0.9	0.8	0.1	Increase
Fiber, g/day	25	27	17	Increase
Cholesterol, g/day	339	338	293	None
*Food intake*				
Virgin olive oil, g/day	50	32	Not Available	Increase
Refined olive oil, g/day	0.9	10.3	Not Available	
Nuts, g/day	10	40	11	Increase
Fruit, g/day	401	406	149	Increase
Vegetables, g/day	340	336	246	Increase
Legumes, g/day	22	22	6	Increase
Whole grains, g/day	27	28	28	None
Refined grains, g/day	181	178	165	None
Pastry, sweets, g/day	17	16	Not Available	Decrease
Meat, g/day	119	119	118	None
Fish/seafood, g/day	101	103	17	Increase
Dairy, g/day	366	370	399	None

Characteristics of average US diet gathered from NHANES 2011–2012 data as compared to PREDIMED MedDiet participant’s diets. Used with permission from Jacobs Jr. et al. [98].
